# New Noncovalent Inhibitors of Penicillin-Binding Proteins from Penicillin-Resistant Bacteria

**DOI:** 10.1371/journal.pone.0019418

**Published:** 2011-05-09

**Authors:** Samo Turk, Olivier Verlaine, Thomas Gerards, Matej Živec, Jan Humljan, Izidor Sosič, Ana Amoroso, Astrid Zervosen, André Luxen, Bernard Joris, Stanislav Gobec

**Affiliations:** 1 Faculty of Pharmacy, University of Ljubljana, Ljubljana, Slovenia; 2 Centre for Protein Engineering, University of Liège, Liège, Belgium; 3 Laboratory of Organic Chemistry, University of Liège, Liège, Belgium; 4 Lek Pharmaceuticals d.d., Mengeš, Slovenia; Aston University, United Kingdom

## Abstract

**Background:**

Penicillin-binding proteins (PBPs) are well known and validated targets for antibacterial therapy. The most important clinically used inhibitors of PBPs β-lactams inhibit transpeptidase activity of PBPs by forming a covalent penicilloyl-enzyme complex that blocks the normal transpeptidation reaction; this finally results in bacterial death. In some resistant bacteria the resistance is acquired by active-site distortion of PBPs, which lowers their acylation efficiency for β-lactams. To address this problem we focused our attention to discovery of novel noncovalent inhibitors of PBPs.

**Methodology/Principal Findings:**

Our in-house bank of compounds was screened for inhibition of three PBPs from resistant bacteria: PBP2a from Methicillin-resistant *Staphylococcus aureus* (MRSA), PBP2x from *Streptococcus pneumoniae* strain 5204, and PBP5fm from *Enterococcus faecium* strain D63r. Initial hit inhibitor obtained by screening was then used as a starting point for computational similarity searching for structurally related compounds and several new noncovalent inhibitors were discovered. Two compounds had promising inhibitory activities of both PBP2a and PBP2x 5204, and good in-vitro antibacterial activities against a panel of Gram-positive bacterial strains.

**Conclusions:**

We found new noncovalent inhibitors of PBPs which represent important starting points for development of more potent inhibitors of PBPs that can target penicillin-resistant bacteria.

## Introduction

β-lactams (penicillins, cephalosporins and carbapenems) are the most widely used antibiotics, due to their high effectiveness, low cost, ease of use, and minimal side effects. At the molecular level, β-lactams target the transpeptidase activity of penicillin-binding proteins (PBPs) that are involved in bacterial cell-wall biosynthesis[1]. In the presence of these antibiotics, the PBPs form a lethal covalent penicilloyl-enzyme complex that blocks the normal transpeptidation reaction; this finally results in bacterial death. However, Gram-negative bacteria have acquired resistance to β-lactams mainly through three different strategies: production of a specific β-lactam hydrolase (the wide-spread β-lactamases); presence of low-affinity PBPs; and active expulsion of β-lactams via efflux pumps[2]. There is thus an urgent need to develop new antibiotics to overcome the challenge of bacterial resistance to existing antimicrobials.

Methicillin-resistant *Staphylococcus aureus* (MRSA) is a leading cause of hospital- and community-acquired bacterial infection, and is a global health threat[3], [4]. Methicillin resistance in MRSA strains has arisen from acquisition of the *mec*A gene, which encodes a novel β-lactam-insensitive PBP (PBP2a)[5]. The crystal structure of PBP2a in both its apo form and complexed to β-lactams has shown that methicillin resistance is achieved through a distorted active site, which requires an energetically costly β3 strand movement to allow acylation by β-lactam antibiotics[6]. One of the possibilities to overcome this intrinsic poor acylation efficiency of PBP2a is to design new β-lactams that have improved binding affinities due to increased noncovalent interactions between the inhibitor and the active site. On the other hand, noncovalent compounds that bind tightly to the active site without acylation might also provide highly effective inhibitors. Noncovalent inhibitors will not require the unfavorable conformational changes in the active site of PBP2a that are required for acylation, and they will hopefully also not be susceptible to β-lactamases[1], [6]. To date, only a few noncovalent inhibitors of PBPs have been described[7]–[9], and so we screened our in-house bank of compounds for potential inhibition of this important drug target.

## Results and Discussion

**Figure 1:**
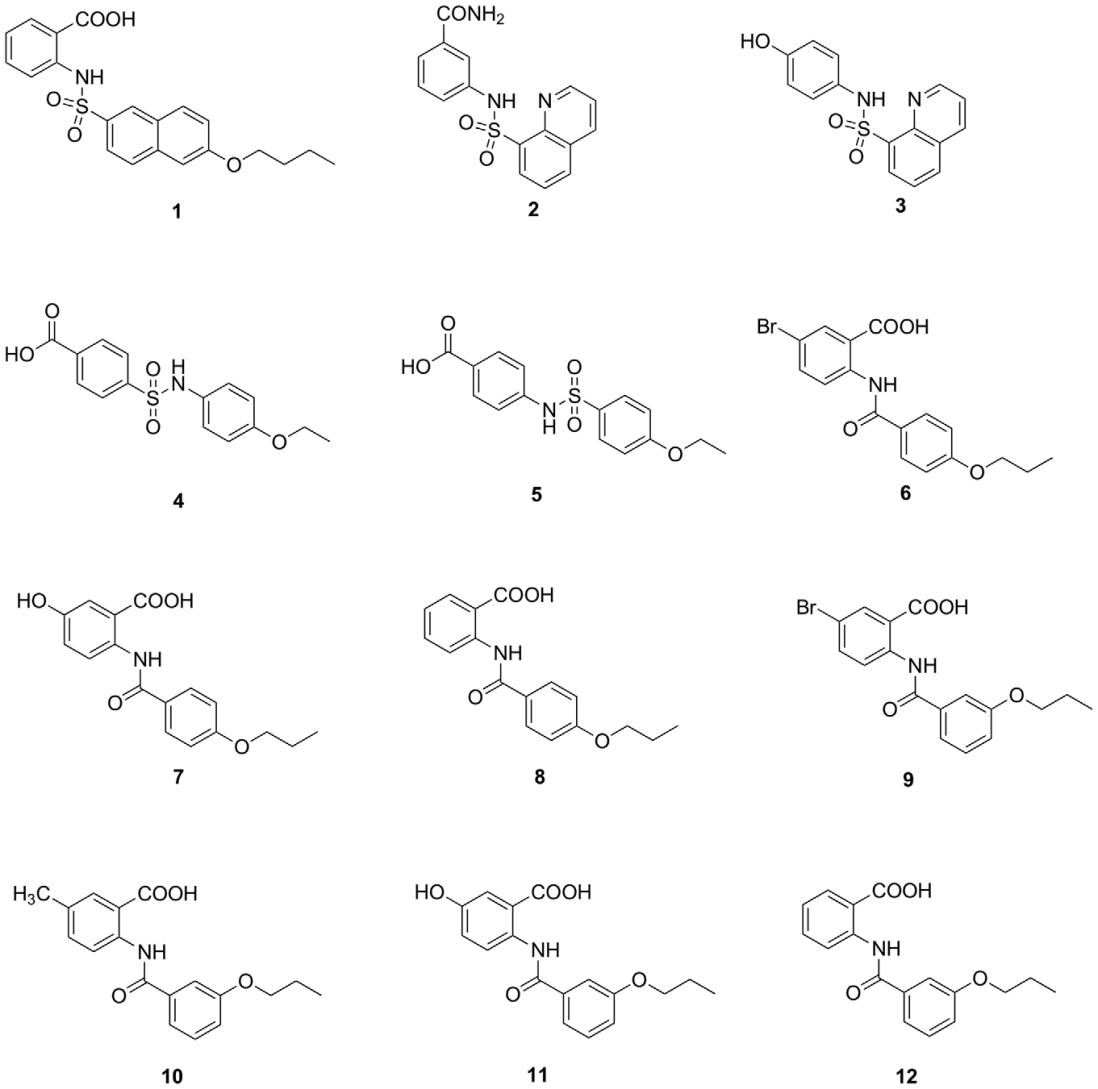
Structures of compounds 1–12.

**Figure 2:**
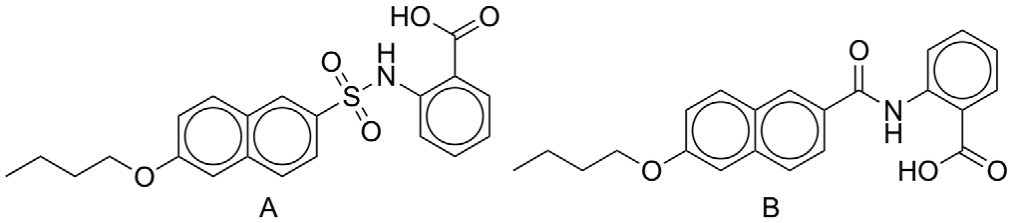
Query A and query B used for computational similarity search.

Screening with a series of more than 250 compounds belonging to different nonreactive chemical classes allowed us to identify an initial hit in compound **1** (Figure 1), which inhibited PBP2a with a promising IC_50_ of 97 µM (Table 1). To obtain a small focused library of structurally related compounds for further studies, computational similarity searches were performed based on the structure of compound **1** as a starting point and using the ChemBridge bank of compounds. The ZINC[10] built-in search engine together with Ftrees 1.0 (BioSolveIT GmbH) software[11] was used. Two different queries were used (query **A** (compound 1) and query **B**, Figure 2) and Tanimoto similarity coefficient was set to 0.90. The only difference between the queries is the bond linking naphthalene ring with anthranilic acid: sulfonamide in query **A** is replaced with an amide in query **B**. From the hits of similarity search we selected only the compounds with unprotected functional groups. Similarity search with FTrees gave compounds **2** and **3** while the rest were obtained by similarity search with ZINC built-in search engine (Figure 1).

Additionally to screening on PBP2a, all of these compounds were also evaluated for inhibition of two other PBPs: PBP2x from the highly drug-resistant *Streptococcus pneumoniae* strain 5204[12], and PBP5fm from the drug-resistant *Enterococcus faecium* strain D63r (Table 1) [13]. All three of the enzymes used for screening are resistant PBPs from important human-pathogen species that are related to community and nosocomial infections, and that therefore represent important drug targets. Similar to PBP2a, resistance to penicillin in PBP2x 5204 and PBP5fm is acquired by active-site distortion, which lowers their acylation efficiency for β-lactams.

**Table 1:** New inhibitors of PBPs from resistant bacteria.^a^

Compound	PBP2a RA [%] (IC_50_)	PBP2x5204 RA [%] (IC_50_)	PBP5fm RA [%] (IC_50_)
1	0 (97 µM)	38^b^ (391 µM)	100
2	58	123	39 (930 µM)
3	67	80	65
4	83	101	100
5	86	81	73
6	0 (210 µM)	41	68
7	74	65	72
8	60	103	74
9	0 (230 µM)	8^b^ (155 µM)	72
10	17 (680 µM)	121	69
11	70	118	61
12	47 (910 µM)	97	34 (>1 mM)

a The data represent mean values of three independent experiments. Standard deviations were within ±10% of these mean values. RA = residual activity of the enzyme at 1 mM inhibitor, unless stated otherwise. IC_50_-values were determined in the presence of 0.01% Triton X-100. ^b^Residual activity of the enzyme at 500 µM inhibitor.

All of these compounds were evaluated biochemically in the presence of detergent (Triton X-100), to exclude the formation of detergent-sensitive promiscuous aggregates[14]. Identical results were observed after 30 or 240 minutes of pre-incubation of the enzyme with the compounds, suggesting rapid and noncovalent inhibition. For the compounds that inhibited at least one of the enzymes under investigation, in-vitro antibacterial activity was determined using a panel of five Gram-negative and 15 Gram-positive bacterial strains (Table 2).

**Table 2:** *In-vitro* antibacterial activities of inhibitors of PBPs from resistant species.

Bacterial Strain	MIC (µg/mL)						
	1	2	6	9	10	12	Ampicillin
*Escherichia coli* ATCC 8739	>1024	>512	>512	>512	>512	>512	4
*Proteus mirabilis* ATCC 29936	>1024	>512	>512	>512	>512	>512	2
*Citrobacter freundii* ATCC 8090	>1024	>512	>512	>512	>512	>512	128
*Pseudomonas aeruginosa* ATCC 27853	>1024	>512	>512	>512	>512	>512	>1024
*Klebsiella pneumoniae* ATCC 13883	>1024	>512	>512	>512	>512	>512	256
*Micrococcus luteus* ATCC 9341	2	>512	32	16	256	256	0.5
*Listeria innocua* ATCC 33090	16	>512	64	16	256	>512	0.25
*Listeria monocytogenes* ATCC 14780	16	>512	64	16	32	>512	0.5
*Bacillus subtilis* ATCC 6633	2	>512	128	32	256	>512	0.5
*Enterococcus faecalis* ATCC 7937	16	>512	64	64	256	>512	2
*Enterococcus faecalis* ATCC 29212	32	>512	32	64	256	256	2
*Enterococcus faecium* ATCC 19434	64	>512	256	16	128	>512	64
*Enterococcus hirae* ATCC 8790	16	>512	32	16	128	256	64
*Streptococcus pneumoniae* ATCC 49619	1	>512	32	1	2	256	0.06
*Streptococcus pneumoniae* ATCC 33400	1	>512	64	1	2	256	0.03
*Streptococcus pneumoniae* D39	1	>512	16	1	2	256	0.06
*Staphylococcus epidermidis* ATCC 12228	32	>512	16	8	128	256	16
*Staphylococcus aureus* ATCC 25923	32	>512	16	32	128	512	0.25
*Staphylococcus aureus* ATCC 43300 (MRSA)	32	>512	128	32	256	512	1024
*Staphylococcus aureus* mp 1 (inducible MRSA)	32	>512	128	32	256	512	1024

As with PBP2a, compound **1** inhibited PBP2x 5204 with an IC_50_ value of 391 µM. The importance of this hit compound is further underlined by its very promising minimum inhibitory concentrations (MICs) against several Gram-positive bacterial strains, including MRSA (32 µg/mL). Indeed, this MIC value for all of the *Staphylococcus aureus* tested strains (32 µg/ml or 80 µM) is lower than the IC_50_ value observed for PBP2a (97 µM), suggesting that *in vivo* compound **1** does not only inhibit PBP2a but could be active on other different cellular targets. This assumption is confirmed by the fact that for *S. aureus* ATCC25923 strain, sensitive to penicillin and devoid of PBP2a, compound **1** has the same MIC value as for the other two resistant *S. aureus* strains (Table 2) where PBP2a is present. To better understand the antibacterial activity of compound **1**, we performed an experiment to evaluate the effect of compound **1** on the protoplasts of *S. aureus* ATCC43300 resistant strain, at a concentration equivalent to 4 times the MIC. After ten minutes, the complete lysis of protoplasts was observed, strongly suggesting an effect of compound **1** on *S. aureus* plasma membranes. Furthermore, killing curves experiments, at equivalent concentration of compound **1**, showed an immediate decrease in the bacterial count, and no viable cells were observed after 120 min, showing a fast bactericidal effect (data not shown) presumably not solely related to the inactivation of PBPs. This suggests that compound **1** may trigger additional cellular events which positively contribute to the antibacterial activity. Further analysis is needed to better understand the precise mode of action of these inhibitors on the bacterial cell.

From the series of sulfonamide compounds **2–5**, the only inhibitor was the quinoline-8-sulfonamide derivative compound **2**, which inhibited PBP5fm. Although the IC_50_ was moderate and compound **2** did not have significant antibacterial activity (MICs above 512 µg/mL), it represents a very important hit compound, as to the best of our knowledge, this is the first noncovalent inhibitor of PBP5fm to be described. Promising inhibitors were also seen in the series of anthranilic acid derivatives, compounds **6–12**. 5-Bromo-2-(4-propoxybenzamido) benzoic acid, compound **6**, was a good inhibitor of PBP2a (IC_50_, 210 µM) with lower inhibition seen for the other two enzymes, and a generally good in-vitro antimicrobial activity against Gram-positive bacteria, even if its growth inhibition of MRSA strains was only moderate. If the 5-bromo substituent was replaced by a 5-hydroxy group or removed, the enzyme inhibitory activity of compounds **7** and **8**, respectively, significantly decreased. In compounds **9–12**, the propoxy-substituent of the benzamido part of the inhibitors is at position 3. 5-Bromo-2-(3-propoxybenzamido)benzoic acid, compound **9**, was a very promising inhibitor of both PBP2a and PBP2x 5204 (IC_50_, 230 µM and 155 µM, respectively). Also, compound **9** showed good antibacterial activity against Gram-positive bacterial strains, and in particular against *E. faecium* and *S. pneumoniae*, as well as both sensitive and resistant *S. aureus.* If the 5-bromo substituent was replaced by a methyl, compound **10**, inhibition of PBP2a was reduced three-fold and inhibition of PBP2x 5204 was lost.

Consequently, the antibacterial activity of compound **10** decreased for all of the bacteria, with the exception of pneumococcal strains, where surprisingly it remained unchanged. Introduction of the hydroxyl group to position 5 in compound **11** further reduced the PBP inhibitory activity. The 5-unsubstituted anthranilic acid derivative compound **12** was a moderate inhibitor of PBP2a and PBP5fm (IC_50_, 910 µM and >1 mM, respectively). The MICs were also consistent with poor PBP inhibitory activity, as they were not better than 256 µg/mL for all of the bacterial strains under investigation.

**Figure 3:**
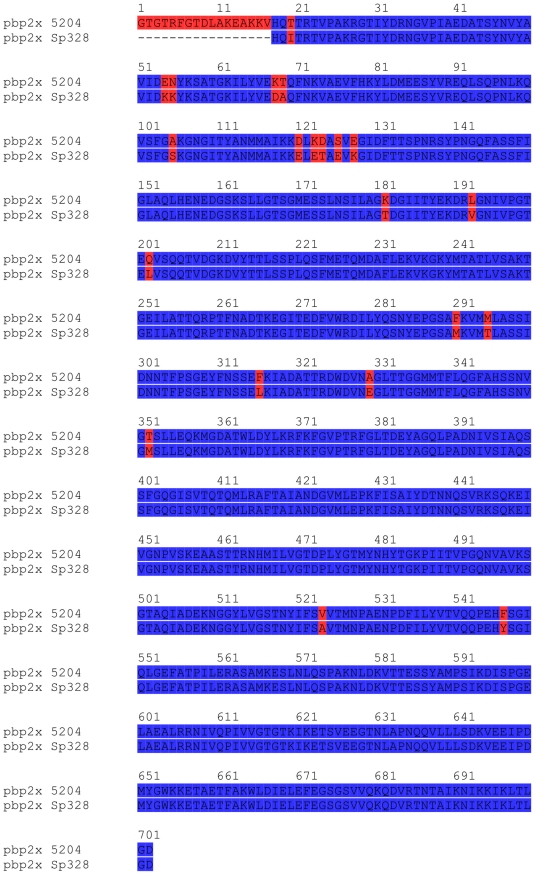
Sequence identity between PBP2x Sp328 and PBP2x 5204. PBP2x Sp328 and PBP2x 5204 show high sequence identity with differences in only few places.

To hypothesize the binding modes of the two best inhibitors, which could assist further structural optimization, inhibitors **1** and **9** were docked into the active sites of PBP2a (pdb code 1VQQ) and PBP2x, respectively. Since there is no known crystal structure of PBP2x 5204, the coordinates of the structurally related (97% sequence identity, Figure 3) PBP2x Sp328 (pdb code 1K25) were used as a template for building the model of PBP2x 5204. Sybyl 8.0 (Tripos Inc.) was used to replace amino acid residues that differ between PBP2x Sp328 and PBP2x 5204. The homology built model was then minimized so that there were no clashes between side chains of the protein.

**Figure 4:**
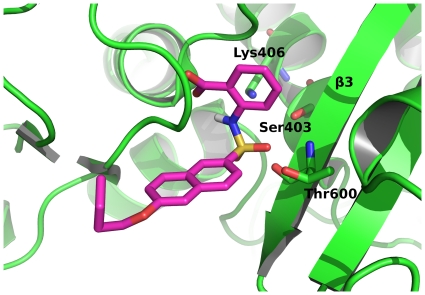
Docking of the sulfonamide inhibitor 1. Inhibitor **1** (magenta) docked into the active site of PBP2a (pdb code 1vqq). The amino acids that form interactions with inhibitor **1** are shown as green sticks.

The docking study was performed using FlexX 3.0[15]. The PBP2a active site contains a nucleophilic Ser403, while the backbone nitrogens of Ser403 and Thr600 form a conserved oxyanion hole, and Lys406 functions as a catalytic base[6]. These data were used to define the size and position of the active-site pocket in the docking experiments. The active site was defined as the area within 10 Å from Lys406. Figure 4 shows the predicted binding conformation of inhibitor **1** in the active site of PBP2a. Inhibitor **1** forms interactions with amino acids that have previously been shown to be important for the binding of the substrate[6]. The sulfonamide oxygen forms H-bonds with Thr600, while the anthranilic acid phenyl ring binds to Lys406 through π-cation interactions. The naphthalene ring forms hydrophobic interactions with Met641 and Tyr446 (not shown, for clarity).

**Figure 5:**
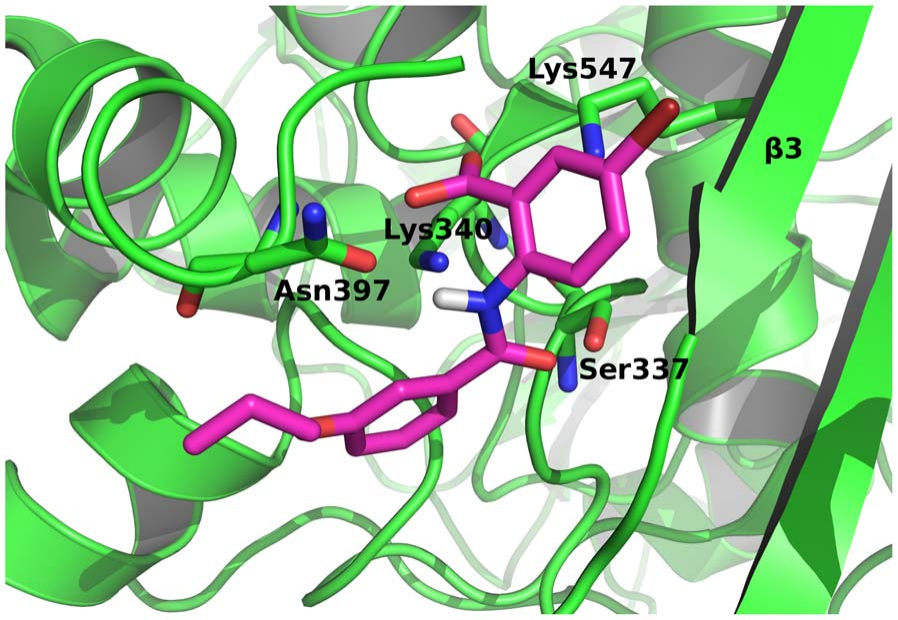
Docking of the anthranilic acid derivative inhibitor 9. Inhibitor **9** (magenta) docked into the active site of PBP2x 5204. The amino acids that form interactions with inhibitor **9** are shown as green sticks.

Similarly, PBP2x 5204 also contains a nucleophilic serine (Ser337) in its active site, as well as two lysines (Lys340 and Lys547) that can act as a catalytic base[16]. Active site was defined as the area within 10 Å from Lys340. Figure 5 shows the predicted binding conformation of inhibitor **9** in the active site of PBP2x 5204. At least two H-bonds are possible: Ser337 and Asn397 form H-bonds with the inhibitor **9** amide oxygen and nitrogen, respectively. Both of the lysines of the active site form interactions with the free carboxylic group of inhibitor **9**. The binding affinity here might be improved by introducing substituents to the position 5 of the anthranilic acid ring. For example, the bulky bromine can be substituted by larger hydrophobic groups, such as isopropyl or tert-butyl. In addition, by comparing the activity of compounds **6** and **9**, which differ only in the position of the propyloxy substituents, we can postulate that the proper position of this substituent on benzoic acid ring appears to be position 3. This is in agreement with our docking study, where unfavorable steric clashes of 4-propyloxy group with the active site of PBP2x 5204 are possible in the case of the compound **6**.

In conclusion, this screening of our in-house bank of compounds followed by similarity searches performed on the ChemBridge databank containing more than 800,000 compounds has led to the identification of new noncovalent inhibitors of PBPs (PBP2a, PBP2x 5204 and PBP5fm) from the penicillin-resistant bacterial strains. Inhibitors **1** and **9** are shown to have promising inhibitory activities of both PBP2a and PBP2x 5204, and good in-vitro antibacterial activities against a panel of Gram-positive bacterial strains. Therefore, inhibitors **1** and **9** represent important starting points for synthetic modifications and development into more potent noncovalent inhibitors of PBPs that can target penicillin-resistant bacteria.

## Materials and Methods

### Computational

Computational part was done on workstation with 4 dual core AMD Opteron 2,0 GHz processors, 16 GB of RAM, 4 320 GB hard drives in RAID10 array and nVidia GeForce 7900 graphic card. Workstation has Fedora 7 64 bit installed.

Similarity searching was done with ZINC [10] built-in engine and with FTrees 1.0 (BioSolveIT GmbH) [11]. Queries A and B were used with both search engines. In all cases ChemBridge bank of compounds was used and Tanimoto similarity coefficient was set to 0.90. Additionally Dynamic Match Search and Global Gap Penalty were user for FTrees search.

Homology building was done with Sybyl 8.0 (Tripos Inc.). Coordinates of PBP2x Sp328 (pdb code 1K25) were used as a template for building the model of PBP2x 5204. Amino acid residues that differ between the two PBPs were replaced. Replaced amino acids were then minimized using Tripos Force Field.

Docking was done with FlexX 3.0 (BioSolveIT GmbH)[15]. For docking in PBP2a we used crystal structure 1VQQ and for docking in PBP2x 5204 we used our homology built model. Active site was defined as the area within 10 Å from Lys406 and Lys340 for PBP2a and PBP2x 5204 respectively. Docking parameters were the same in both cases. For base placement Triangle Matching was used and the program generated maximally 200 solutions per iteration and 200 per fragmentation.

### Chemistry

#### General

Chemicals were from Sigma-Aldrich and Acros Organics, and were used without further purification. Solvents were used without purification or drying, unless otherwise stated. Analytical TLC was performed on Merck silica gel (60F_254_) plates (0.25 mm), and the compounds were visualized with ultraviolet light. Column chromatography was carried out on silica gel 60 (particle size, 240–400 mesh). Melting points were determined on a Reichert hot-stage microscope and are uncorrected. ^1^H-NMR spectra were recorded on a Bruker AVANCE DPX_300_ spectrometer in CDCl_3_ or DMSO-*d_6_* solution, with TMS as the internal standard. IR spectra were obtained on a Perkin-Elmer 1600 FT-IR spectrometer. Microanalyses were performed on a Perkin-Elmer C, H, N analyzer 240 C. Mass spectra were obtained using a VG-Analytical Autospec Q mass spectrometer.

#### Synthesis of compound 1

**Figure 6:**
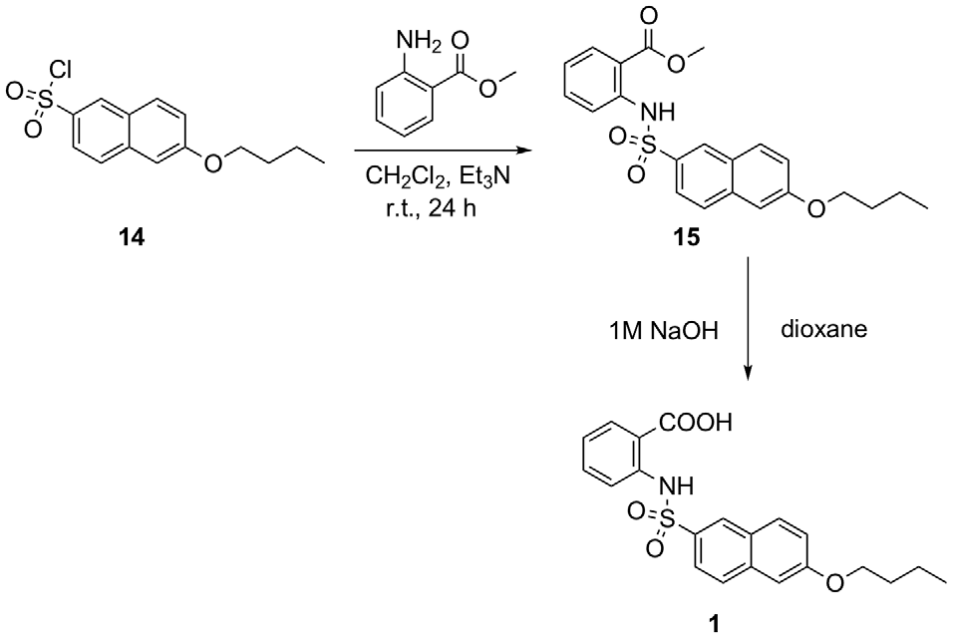
Synthesis of inhibitor 1.

6-Butoxynaphthalenesulfonyl chloride **14** was prepared according to the three-step procedure previously described (Scheme 1)[17]. In the next step it was coupled with methyl anthranilate to give sulfonamide **15**. The target compound **1** was obtained after the final hydrolysis with 1 M NaOH/dioxane (Figure 6).

#### Procedure for the preparation of methyl 2-(6-butoxynaphthalene-2-sulfonamido)benzoate (15)

To an ice-cold solution of methyl anthranilate (2.04 mmol, 309.4 mg) in dry CH_2_Cl_2_ (5 mL), Et_3_N (2.5 mmol, 505 mg) and a solution of compound **14** (1.0 mmol, 597.0 mg) in CH_2_Cl_2_ (5 mL) were slowly added. The reaction mixture was stirred at 0°C for 30 min and then at room temperature for 24 h. After the reaction was complete (monitored by TLC), 1 M HCl (15 mL) was added and the mixture extracted with CH_2_Cl_2_ (3×20 mL). The combined organic layers were washed with brine (2×30 mL), dried over Na_2_SO_4_, filtered, and evaporated under reduced pressure. The residue was purified by silica gel column chromatography (EtOAc-hexane = 1/3), to provide a white solid, 310 mg (75%), which was immediately used in the next step.

#### Procedure for the preparation of 2-(6-butoxynaphthalene-2-sulfonamido)benzoic acid (1)

To a stirred solution of **15** (0.60 mmol, 250.0 mg) in dioxane (5 mL), 1 M NaOH (3 mL) was slowly added, and the reaction mixture stirred overnight at room temperature. Then the reaction mixture was diluted with H_2_O (20 mL) and washed with EtOAc (3×20 mL). The aqueous phase was acidified with 1 M HCl to pH 1, and extracted with EtOAc (3×20 mL). The combined organic layers were washed with brine (2×30 mL), dried over Na_2_SO_4_, filtered, and then the solvent was removed under reduced pressure, to yield a white solid, 213 mg (85%). Mp = 192–194°C; ^1^H-NMR (300 MHz, CDCl_3_): *δ* ppm 0.94 (t, *J* = 7.5 Hz, 3H, -CH_2_C*H*_3_); 1.40–1.52 (m, 2H, -OCH_2_CH_2_C*H*_2_CH_3_); 1.71–1.80 (m, 2H, -OCH_2_C*H*_2_CH_2_CH_3_); 4.11 (t, *J* = 6.6 Hz, 2H, -OC*H*_2_); 7.05–7.10 (m, 1H, Ar-*H*); 7.28 (dd, *J* = 9.0, 2.4 Hz, 1H, Ar-*H*); 7.41 (d, *J* = 2.4 Hz, 1H, Ar-*H*); 7.48–7.58 (m, 2H, Ar-*H*); 7.69 (dd, *J* = 8.7, 2.1 Hz, 2H, Ar-*H*); 7.85–7.94 (m, 2H, Ar-*H*); 8.05 (d, *J* = 9.0 Hz, 1H, Ar-*H*); 8.49 (d, *J* = 1.8 Hz, 1H, Ar-*H*); 11.18 (br s, 1H, -SO_2_N*H*-); 13.95 (br s, 1H, -COO*H*); EI-MS: m/z 399 (C_21_H_21_NO_5_S, M^+^); CHN found C: 63.45, H: 5.50, N: 3.51; calc. for C_21_H_21_NO_5_S C: 63.14, H: 5.30, N: 3.51.

#### Purity of compounds 2–12

For compounds **2**, **3**, **5**, **7**, **8**, **11**, and **12**, HPLC purity was determined using an Agilent Eclipse C18 column (4.6×50 mm, 5 µm) with a flow rate of 1.0 mL/min, detection at 254 nm, and an eluent system of: A = H_2_O with 0.1% TFA; B = MeOH. The following gradient was applied: 0–3 min, 30% B; 3–18 min, 30% B→80% B; 18–23 min, 80% B; 23–30 min, 80% B→30% B; run time = 30 min; T = 25°C.

3-(Quinoline-8-sulfonamido)benzamide (**2**): Retention time: 5.59 min, HPLC purity: 99.06%.

*N*-(4-Hydroxyphenyl)quinoline-8-sulfonamide (**3**): Retention time: 11.20 min, HPLC purity: 96.12%.

4-(4-Ethoxyphenylsulfonamido)benzoic acid (**5**): Retention time: 13.86 min, HPLC purity: 99.56%.

5-Hydroxy-2-(4-propoxybenzamido)benzoic acid (**7**): Retention time: 19.65 min, HPLC purity: 100.0%.

2-(4-Propoxybenzamido)benzoic acid (**8**): Retention time: 22.56 min, HPLC purity: 98.62%.

5-Hydroxy-2-(3-propoxybenzamido)benzoic acid (**11**): Retention time: 19.96 min, HPLC purity: 99.38%.

2-(3-Propoxybenzamido)benzoic acid (**12**): Retention time: 22.92 min, HPLC purity: 99.41%.

Purity for compounds **4**, **6**, **9** and **10** was determined with elemental analysis.

4-(*N*-(4-Ethoxyphenyl)sulfamoyl)benzoic acid (**4**): CHN found C: 55.94, H: 4.79, N: 4.50; calc. for C_15_H_15_NO_5_S C: 56.06, H: 4.70, N: 4.36.

5-Bromo-2-(4-propoxybenzamido)benzoic acid (**6**): CHN found C: 54.01, H: 4.13, N: 3.77; calc. for C_17_H_16_BrNO_4_ C: 53.99, H: 4.26, N: 3.70.

5-Bromo-2-(3-propoxybenzamido)benzoic acid (**9**): CHN found C: 53.66, H: 4.22, N: 4.03; calc. for C_17_H_16_BrNO_4_ C: 53.99, H: 4.26, N: 3.70.

5-Methyl-2-(3-propoxybenzamido)benzoic acid (**10**): CHN found C: 68.99, H: 5.96, N: 4.51; calc. for C_18_H_19_NO_4_ C: 68.99, H: 6.11, N: 4.47.

### Biology

#### Enzymatic inhibition assays for low affinity PBP2a and PBP5fm

PBP2a from *Staphylococcus aureus* ATCC 43300 and PBP5fm from *Enterococcus faecium* D63r were overproduced and purified as described previously[13], [18].

Each of the purified PBPs (2.5 µM) were first incubated with 1 mM potential inhibitor in 100 mM phosphate buffer, 0.01% Triton X-100[19], pH 7, for 4 h at 30°C. Then, 25 µM fluorescein-labeled ampicillin[20] was added to detect the residual penicillin binding activity (RA). The samples were further incubated for 30 min at 37°C in a total volume of 20 µL. Denaturation buffer was added (0.1 M Tris/HCl, pH 6.8, containing 25% glycerol, 2% SDS, 20% β-mercaptoethanol and 0.02% bromophenol blue) and the samples were heated to 100°C for 1 min. The samples were then loaded onto a 10% SDS-acrilamide gel (10×7 cm) and electrophoresis was performed for 45 min at 180 V (12 mA). Detection and quantification of the RAs were with Molecular Image FX equipment and Quantity One software (BioRad, Hercules, CA, USA). Three independent experiments were carried out for each inhibitor.

#### Enzymatic inhibition assays for PBP2x 5204

PBP2x-5204 from *Streptococcus pneumonia* [12] and *N*-benzoyl-D-alanyl-thioglycolate S2d[21], [22] were prepared as previously described. All assays with PBP2x 5204 were carried out in 96-well microtiter plates (Brand, Wertheim, Germany). PBP2x 5204 (0.6 µM) was incubated in the presence of the potential inhibitors in 10 mM sodium phosphate buffer (pH 7.0) containing 70 mM D-alanine and 0.01 mg/mL BSA, for 4 h at 25°C. This preincubation was used to also detect slow binding inhibitors. After the preincubation, the RA of PBP2x 5204 was determined. The initial rate of hydrolysis of 1 mM S2d in the presence of 1 mM DTNB was determined by monitoring the increase in absorbance at 412 nm (DTNB: ε[Δ ε] = 13600 M^−1^ s^−1^) using a microplate absorbance reader (Power Wave X, Biotek Instruments, Winooski, U.S.A.). The rate of spontaneous hydrolysis of S2d in the presence of the potential inhibitors was also determined in the absence of PBP2x 5204. All of the assays were carried out in triplicate. The determination of the RA of PBP2x 5204 in the absence of inhibitors was carried out six times on each plate. A test compound was considered as an inhibitor if the RA was <80%. In this case, to reveal false positives, the assays were also carried out under the same conditions in the presence of 0.01% Triton X-100. As described in the literature, promiscuous inhibitors (false positives) are slow binding, noncompetitive inhibitors. To avoid detailed kinetic investigations[8], it is possible to identify such compounds by carrying out the assays in the presence of Triton X-100[14], [19]. Promiscuous inhibitors show no inhibition in the presence of Triton X-100. The IC_50_ values in the presence of Triton X-100 were determined if the RAs were <50%. (RA: ∼50%, IC_50_∼c, c: concentration of compound in the assay; RA >50%, IC_50_ > c). The RA was measured over a range of concentrations, from which the IC_50_ values were determined by non-linear regression analysis, using Sigma Plot (Systat software) and fitting the data to the equation *y* = *y*0+(*a*×*b*)/(*b*+*x*)[23].

#### Antibacterial activity

Determination of the antibacterial activities was carried out on microtiter plates, in 200 µL (final volume) of Müeller-Hinton Broth (MHB), following EUCAST (European Committee on Antimicrobial Susceptibility testing)/CLSI (Clinical and Laboratory Standard Institute) recommended procedures[24], [25]. The compounds were solubilized in MHB, just before use. Inocula were prepared for each strain by resuspending isolated colonies from 18 h cultured plates. Equivalents of 0.5 Mac Farland turbidity standards (approximately 1×10^8^ CFU mL^−1^) were prepared in saline solution (0.085% NaCl) and then diluted 200-fold in MBH. MICs were determined as the lowest dilution of product that showed no visual turbidity.

## References

[1] Macheboeuf P, Contreras-Martel C, Job V, Dideberg O, Dessen A (2006). Penicillin binding proteins: key players in bacterial cell cycle and drug resistance processes.. FEMS Microbiol Rev.

[2] Wilke MS, Lovering AL, Strynadka NCY (2005). Beta-lactam antibiotic resistance: a current structural perspective.. Curr Opin Microbiol.

[3] Chambers HF (1997). Methicillin resistance in staphylococci: molecular and biochemical basis and clinical implications.. Clin Microbiol Rev.

[4] Enright MC, Robinson DA, Randle G, Feil EJ, Grundmann H (2002). The evolutionary history of methicillin-resistant Staphylococcus aureus (MRSA).. Proc Natl Acad Sci U S A.

[5] Wu SW, de Lencastre H, Tomasz A (2000). Recruitment of the mecA gene homologue of Staphylococcus sciuri into a resistance determinant and expression of the resistant phenotype in Staphylococcus aureus.. J Bacteriol.

[6] Lim D, Strynadka N (2002). Structural basis for the beta lactam resistance of PBP2a from methicillin-resistant Staphylococcus aureus.. Nat Struct Biol.

[7] Toney JH, Hammond GG, Leiting B, Pryor KA, Wu JK (1998). Soluble penicillin-binding protein 2a: beta-lactam binding and inhibition by non-beta-lactams using a 96-well format.. Anal Biochem.

[8] Zervosen A, Lu WP, Chen Z, White RE, Demuth TP (2004). Interactions between penicillin-binding proteins (PBPs) and two novel classes of PBP inhibitors arylalkylidene rhodanines and arylalkylidene iminothiazolidin-4-ones.. Antimicrob Agents Chemother.

[9] Miguet L, Zervosen A, Gerards T, Pasha FA, Luxen A (2009). Discovery of new inhibitors of resistant Streptococcus pneumoniae penicillin binding protein (PBP) 2x by structure-based virtual screening.. J Med Chem.

[10] Irwin JJ, Shoichet BK (2005). ZINC − A free database of commercially available compounds for virtual screening.. J Chem Inf Model.

[11] Rarey M, Stahl M (2001). Similarity searching in large combinatorial chemistry spaces.. J Comput Aid Mol Des.

[12] Carapito R, Chesnel L, Vernet T, Zapun A (2005). Pneumococcal beta-lactam resistance due to a conformational change in penicillin-binding protein 2x.. J Biol Chem.

[13] Sauvage E, Kerff F, Fonzé E, Herman R, Schoot B (2002). The 2.4-Å crystal structure of the penicillin-resistant penicillin-binding protein PBP5fm from Enterococcus faecium in complex with benzylpenicillin.. Cell Mol Life Sci.

[14] Shoichet B (2006). Screening in a spirit haunted world.. Drug Discovery Today.

[15] Rarey M, Kramer B, Lengauer T, Klebe G (1996). A fast flexible docking method using an incremental construction algorithm.. J Mol Biol.

[16] Dessen A, Mouz N, Gordon E, Hopkins J, Dideberg O (2001). Crystal structure of PBP2x from a highly penicillin-resistant Streptococcus pneumoniae clinical isolate: a mosaic framework containing 83 mutations.. J Biol Chem.

[17] Humljan J, Kotnik M, Contreras-Martel C, Blanot D, Urleb U (2008). Novel naphthalene-N-sulfonyl-D-glutamic acid derivatives as inhibitors of MurD a key peptidoglycan biosynthesis enzyme.. J Med Chem.

[18] Lemaire S, Glupczynski Y, Duval V, Joris B, Tulkens PM (2009). Activities of ceftobiprole and other cephalosporins against extracellular and intracellular (THP-1 macrophages and keratinocytes) forms of methicillin-susceptible and methicillin-resistant Staphylococcus aureus.. Antimicrob Agents Chemother.

[19] Feng BY, Shoichet BK (2006). A detergent-based assay for the detection of promiscuous inhibitors.. Nat Protoc.

[20] Lakaye B, Damblon C, Jamin M, Galleni M, Lepage S (1994). Synthesis purification and kinetic properties of fluorescein-labelled penicillins.. Biochem J.

[21] Adam M, Damblon C, Jamin M, Zorzi W, Galleni M (1990). Chromogenic depsipeptide substrates for beta-lactamases and penicillin-sensitive DD-peptidases.. Biochem J.

[22] Schwyzer R, Hurlimann C (1954). Coenzym A: Modellreaktionen zur enzymatischen Aktivierung von Acylderivaten des Coenzyms A.. Helv Chim Acta.

[23] Macheboeuf P, Fischer DS, Brown T, Zervosen A, Luxen A (2007). Structural and mechanistic basis of penicillin-binding protein inhibition by lactivicins.. Nature Chem Biol.

[24] (2009).

[25] (2003).

